# Methods for nutrition monitoring in cancer patients: a cognitive perspective

**DOI:** 10.3332/ecancer.2012.259

**Published:** 2012-06-25

**Authors:** C Lucchiari, M Masiero, G Pravettoni

**Affiliations:** Università degli Studi di Milano, Milan, Italy

**Keywords:** *nutrition*, *cancer*, *decision-making*, *dietary assessment methods*, *personalized approach*

## Abstract

In the present medical context, the evaluation and the monitoring of factors other than mere physical symptoms are an urgent demand. In particular, the issue of quality of life (QoL) has become a relevant target in the treatment of cancer. However, the approach towards these aspects is not well standardized and the actual applications in a concrete setting are fragmented, left to personal or local initiative. If this is true for QoL in general, it is particularly relevant in the specific field of nutrition. Indeed, though the growing awareness of a correlation between chronic diseases and dietary habits has led to an increased interest in nutrition, both before and after cancer, very little is still known about the methods that measure this important variable of the QoL. Indeed, good nutrition may have a relevant impact on QoL, positively affecting both the physical and psychological well-being. Targeting this issue implies using proper instruments to both monitor and educate the patients. Hence, we argue that it is vital for oncologists to be able to individuate the best tool available in a specified context, so as to achieve an important goal with little effort, also adopting standardized strategies proved to be efficacious. In this framework, we briefly reviewed the tools more frequently reported in the scientific literature. We suggest that through a cognitive approach, it is possible to achieve important clinical targets, initially by understanding the patients’ needs, values, and psychosocial factors involved in nutritional behaviour and food-related decisions, in order to develop a personalized approach. Hence, this is the only way to support concrete actions for promoting healthier diets, thus preventing recurrences, monitoring chronic conditions, and supporting a good QoL.

## Introduction

### Nutritional issues and chronic disorders

In 2003, the Food and Agriculture Organization of the United Nations (FAO) and the World Health Organization (WHO) published a pragmatic document on diet and nutrition correlation titled *Diet, Nutrition and the Prevention of Chronic Diseases* [1].

This document stressed the scientific evidence on the relationship between diet, nutrition, and physical activity in the aetiology of chronic diseases. It concluded that a diet low in saturated fats, sugars, and salt and high in vegetables and fruits, together with regular physical activity, has a preventive effect on death caused by cardiovascular diseases, several forms of cancer, diabetes, and obesity. The European Prospective Investigation into Cancer and Nutrition (EPIC) programme [2], aimed to analyse the relationship between lifestyle, context, energy intake, and nutritional behaviour and the incidence of cancer, supports the same conclusions. Hence, we can argue that rapid developments in several scientific fields have highlighted the importance of monitoring nutrition habits in preventing and controlling morbidity and mortality resulting from chronic diseases.

The past 20 years have been characterized by a steady growth of obesity and being overweight in the world’s population. This phenomenon is called the ‘obesity epidemic’ [3]. Obesity has been defined by the WHO as a state of excessive weight that may cause health-related danger. An obesogenic condition is described as being the result of a combination of excessive energy intake and inadequate physical activity [3]. Being overweight, when not pathological, is mostly caused by unhealthy food choices. The obesity epidemic, the increasing occurrence of diseases in the world’s population, and the growing awareness of a correlation between chronic diseases and dietary habits have led to an increased interest in nutrition.

The main reasons for the development of obesity are lifestyle shifting, in historical, social, and environmental transitions. In general, nutritional habits changed during the second half of the twentieth century. After World War II, there were changes in mood, lifestyle, and the time of food consumption in developing countries. Nowadays, food is overly present and available, for example in supermarkets, bars, restaurants, and at home. Furthermore, there are several features of daily meals that have changed: people eat faster and several times a day (at breakfast, brunch, lunch, snack, appetizer, and dinner). Inherent to this new lifestyle is an increase in stress, and, conversely, less energy expenditure, increased consumption of high-calorie foods, and adoption of a sedentary lifestyle.

The relationship between cancer and food intake was already stressed in the 1940s. For instance, during a clinical trial in a sample of rodents, a correlation between caloric restriction and cancer rate reduction was observed [4]. Furthermore, it was found that caloric constraint reduced the development of mammary tumours in a sample of animals. The effects were detected for different tumours [5, 6]. However, it was only in 1981 that the international attention on carcinogenesis and food was spread. In a study on the causes of cancer mortality in the United States, Doll and Peto [7] suggested that dietary factors might account for 35% of cancer deaths [8]. The year 1981 was recognized as a ‘starting point’ for nutrition research. Indeed, it was since then that scientists with different expertise (physicians, biologists, nutritionists, and psychologists) have started to investigate systematically the correlation between diet habits and health disorders.

It has been assessed that 30–40% of all tumours could be prevented adopting a healthy diet [9]. Taking this fact into account, the cancer burden and the increase of obesity in the world’s population have favoured the creation of nutritional issue. Different lifestyles and environmental factors (tobacco and alcohol consumption, food intake, and physical activity) can be described as predictive for the carcinogenesis [10].

This assumption has been tested in both human population studies and laboratory experiments. Renehan [11] observed a positive association between body max index (BMI) and cancer of the thyroid, leukemia, malignant melanoma, non-Hodgkin lymphoma, and multiple myeloma. Bergstrom [12] described a correlation between overweight and prostate cancer; while Lahmann [13] and Shouten [14], and Benson [15] described an association of overweight with ovary cancer and with brain cancer, respectively.

More recently, investigations by the International Agency for Research on Cancer (IARC) and the World Cancer Research Fund (WCRF) have remarked that a major cancer risk could be associated with being overweight [16].

### How does a healthy diet prevent carcinogenesis?

Before being harmful to human beings, malignant cells could be present in a latency form. In this phase, a correct diet (e.g. habitual intakes of fruits, vegetables, grains, and cereals) contrasts healthy cell transformation into malignant cell. Preventive action of the food is achieved through two strategies: reducing the malignant cell development and eradicating the malignant cells (apoptosis), and favouring the development of microenvironments that contrast the survival of the malignant cell [17]. Furthermore, food choice and diet are considered the fundamental factors in survival planning for patients who are recently diagnosed with cancer, patients who are undergoing cancer treatment (radiotherapy, chemotherapy, etc.), and subjects who have survived many years after their early diagnosis and cancer treatment.

According to clinical evidence, diet strongly influences cancer prevention, disease development, treatment tolerance, and cancer recurrence [18]. Generally speaking, cancer care includes several specific stages: the active treatment stage, the recovery stage during which the physical need has to be restored, a health maintenance stage to prevent cancer recurrence and other related diseases, and finally living with advanced cancer. Every stage has specific nutrition needs that must be satisfied [19].

We argue that healthy eating programmes can be organized into three directions. For each direction, it is important to identify an adequate diet assessment method and the correct method to promote health behaviours.

- Prevention nutrition recommendations: These recommendations may promote and disseminate correct lifestyle behaviour within the healthy population. The principal aim of this recommendation is the education about a better lifestyle, for example a better eating choice, physical activity, and reduction of smoking and alcohol consumption. Identifying the attitudes associated with cancer risk is central to prevent not only cancer [20], but also other disorders related to unhealthy lifestyles.
- Nutrition recommendations during therapy: These recommendations improve healthy food intake during the therapy in order to prevent underweight and to avoid disorders related to cancer. Nutrition is an important component of the management of individuals diagnosed with cancer. Regardless of undergoing active therapy, recovering from cancer treatment, or in remission with the aim to avoid recurrence, the potential importance of optimal energy and nutrient intake has been examined. The primary goals are to prevent or reverse nutrient deficiencies, to preserve lean body mass, to minimize nutrition-related side effects, and to maximize the quality of life (QoL). Consistent screening practices to identify patients at risk for malnutrition are essential. Nutrition therapy is an integral part of cancer care from diagnosis through treatment and recovery [21]. For example, a typical nutritional problem that oncology must cope with is the cachexia in patients who are undergoing active treatment. Cachexia refers to an ongoing loss of skeletal muscle mass, and generally leads to functional impairment [22]. Normally, cancer patients experience weight loss (39–82%) and a shift in eating habits (30–80%) [23].

There are many websites that provide advice and guidelines to organize a proper diet during and/or after therapy [24]. For example, The National Cancer Institute published a book on nutrition for cancer patients titled *Eating Hints. Before, During, and After Cancer Treatment*. The goal of this book is to manage the eating problem (appetite loss, shifting in sense of taste and smell, and weight gain or loss related to the side effect of the treatment) and the better diet in cancer patients [25].

Currently, similar booklets, brochures, and pamphlets to manage food-related decisions during/after the therapy are available. They are created to manage different needs and different concrete situations. For instance, the IARC edited different brochures:

- ‘Nutrition of the Cancer Patients’: to evaluate energy intake during the therapy [26]
- ‘Surviving Cancer with Physical Activity’: to manage the energy intake and the physical activity [27]

The issue is to develop a standard tool that may be adapted for a specific real context and particular requirements and be very useful to both health personnel and patients. In particular, future research should address the possibility to give rise to interactive tools to be integrated into electronic personal health records. Furthermore, psychological and cognitive investigations are needed to optimize information delivery and its actual use by outpatients. Indeed, information alone is not sufficient to promote changes in unhealthy habits. Instead, a validated psychological and cognitive approach is needed.

- Recommendations for survivorship: Cancer diagnosis is a ‘teachable moment’, leading survivors to make constructive changes in eating habits and physical activity [28, 29]. Overweight is common during and after treatment for several neoplastic conditions; it enhances risk for functional decline, cancer recurrence, and cancer-related death [30, 31]. These recommendations sustain a correct nutrition in survivorship to decrease the risk of second malignancies and other chronic diseases such as cardiovascular disease [32]. Detailed information about the amount, size, typology, frequency, and physical activity for cancer prevention, treatment, and survivorship are published on http://www.cancer.org/index [33]. Little cognitive research is now available with regard to the relationship between survivorship, QoL, and nutrition, but the issues will surely become central in the next few years.

In general, it is important to note that good nutrition has a relevant impact on QoL. Indeed, a proper diet may positively affect both physical health and psychological well-being.

Hence, considering these points, we argue that it is important to understand how to help healthy people and cancer patients adopt a healthy lifestyle. To achieve this aim and to support the oncologists as well as other experts in managing the patient’s needs, it is necessary to apply the correct dietary assessment method. Dietary assessment methods are valuable tools that permit insights into the decision-making about food consumption and habit evaluation.

### Dietary assessment methods in a clinical setting

Dietary assessment methods have been investigated extensively [34]. We briefly review current dietary instruments that could be used for cancer patients and survivorship. An adequate dietary assessment method is a key requirement when attempting to understand dietary profiles in healthy and cancer populations.

The most frequently used tools are as follows (see Table 1).

***Food Frequency Questionnaires (FFQ)***: It is usually used in the longitudinal studies to assess food habits. It elicits information about frequency of consumption and portion size of food. FFQ includes two sections: one section contains a food checklist, while the other could give the opportunity for the subjects’ comments. Respondents indicate their frequency per day, per week, or per month (in relation with the experimental protocol). Typically, FFQ is used in epidemiologic studies on dietary lifestyle and chronic diseases [35], but it may also be used in clinical settings [36].

It is easy and cheap to administer, and generally it is positively evaluated by respondents [37]. FFQ has been utilized in case-control or cohort research to measure the association between dietary intake and disorder risk [38]. It can be self-administered, and the singular results are acquired through optical scanning or web-based application. In general, while some papers state that it is limited to 100 food categories [39], it may be extended to 132 [35] or 150 [40]. The completion time is between 30 and 60 min. In addition, a short version of the FFQ has been developed between 40 and 60 list items.

Many forms of the FFQ are available for different target populations and with diverse objectives. For instance, the *Dietary History Questionnaire (DHQ)* is a development of FFQ based on cognitive research to be subject-friendly and easily filled in different contexts. It is composed of 124 food items and considers both portion size and dietary supplement. The Fred Hutchinson Cancer Research Center Food Frequency Questionnaire is a good example of a FFQ targeted at cancer patients. The main disadvantage of FFQ-style tools is that they ask respondents to provide ‘typical’ frequencies and portion sizes, generally, over the previous year and requires mental averaging over varying intakes and seasons. These strategies might introduce cognitive biases in the dietary evaluation [41].

***Diet History***: The diet history approach was proposed by Burke and Stuart in 1940. It investigates not only the frequency of food intake, but also the usual make-up of meals. Diet history is characterized by three elements: an interview on habitual eating pattern, a food list that evaluates frequency and the amount of food intake, and a 3-day diet record [42, 43]. The advantage of diet history is that meal patterns and food intake are investigated extensively. In contrast, a disadvantage is that subjects are asked to recall and to report habitual foods and amounts of the food consumed in previous days.

***Healthy Eating Index (HEI)***: Developed in 1995 and revised in 2005, the Healthy Eating Index is one of the many dietary indexes published. In general, the aim of a dietary index is to measure the quality of nutrition even though it is not aimed at assessing the diet in term of energy, micronutrient, and macronutrients. A dietary index is particularly useful for evaluating the diet of a population, but it could also be used as a supportive tool in clinical settings, where clinicians, psychologists, or researchers are interested in collecting standardized data about food patterns.

HEI includes ten items of which five measure the nutrient adequacy of the diet, four measure aspects of the diet that should be limited, and one measures variety in the food choice. Each item is scored on a 0 to 10 scale, resulting in a total standardized score of 100.

Finally, it generates three different results: a total score of more than 80 is considered good; a total score included within the range 51–80 means that respondents require improvements; and a total score of less than 51 is considered poor. The HEI can be used in nutrition monitoring, health interventions, as well as for research [44].

***Food Records or Diaries***: Diaries contain categories and quantities of foods consumed per day. Subjects record the foods and beverages, and their quantities consumed over one or more days. Overall energy intake is recorded with pictures, scales, or other measures. These tools are normally ‘pen and paper’, but electronic versions are now available or are under development, including cell phone and tablet apps that are able to implement real-time and portable monitoring [40]. An advantage of this assessment method is that diaries provide quantitative information about food consumed during the recording time.

There are two main problems inherent to this assessment strategy. First, if you need to gather collective data, for example, for research purposes, you have to consider potential data distortions due to a sampling bias, since subjects’ involvement results in an elevated motivation rendering data not representative for the broad population. Second, you cannot be sure if the diary is actually compiled on a day-by-day basis. Instead, less-motivated respondents should decide to compile the diary once a week or when they are reminded of it, potentially introducing memory-based biases. Indeed, when the recording time increases (more than 7 days), the validity of the recalling decreases. To overcome this bias, an electronic version with a chronological recording tool should be used, signalling when diaries were actually filled in.

***24-Hour Dietary Recalls***: A particular tool to collect data about nutritional behaviour is the 24-hour dietary recalls. Interviews are conducted to gather data. The principal aim of this tool is to record in detail food and beverage intake in the previous 24 hours. A disadvantage of this tool concerns cognitive and motivational biases. For instance, many subjects might find it difficult to distinguish between what they remember eating and what they actually ate a few hours ago. Furthermore, implicit motives could lead the subject to report only some part of their meal, maybe omitting to report less-healthy foods or what they consider that a physician would evaluate as a bad choice [39]. We believe that it is important to stress two benefits of this tool:

- Literacy of the subjects is not required in advance, because an expert interviewer will administer the instrument. As a result of this, the 24-hour dietary recall is useful across a wide range of populations.
- Dietary recall occurs after the food has been consumed, so the dietary assessment will have a minor impact on dietary choice (reactivity). Alternatively, other methods, such as FFQ, generally have a more relevant impact on dietary choice.

Conversely, the main disadvantage is that the subjects may not communicate their food intake correctly for different factors connected to memory, understanding and knowledge, interview condition, and so on. This tool was often used to evaluate the percentage of the population that adopts satisfactory or unsatisfactory diets.

A particular example of a 24-hour dietary recall is the Automated Self-administered 24-hour Recall (ASA24). In this case, the administration of the tool is supported by a computer interface, rather than by a human interviewer. It is constituted by a *respondent website* used to gather and recall and a *research website* used to organize and collect data for later analyses. Subjects are asked to complete a 24-hour recall for the previous day, using a multimedia interface. The subject has to report eating occasions, time, mood (alone or in group, at home, at a restaurant, etc.), and frequency of consumption. The tool requires an exhaustive description about preparation and portion measure. ASA24 was developed in order to provide an animated guide (audio and visual) to enhance practice in low-literacy subjects [45].

*Short Dietary Assessment Instrument*: It is used to evaluate a specific range of food intake instead of the total diet. It assesses, for example, the intake of fruit and vegetable, grains, dairy products, percentage energy from fat, and so on. It is useful in clinical settings and for health promotion programmes. Short dietary assessment instruments are usually used to help individuals change their diet. In contrast to other assessment instruments, short instruments focus on specific eating behaviours. However, they fail to detect information on the whole diet of an individual.

The use of these brief methods may be advantageous for characterising, population’s average intake, discriminating between individuals or populations, analysing relations among food habits and other variables (e.g. sex, age, race, and diet), and to compare data collected in different trials for research purposes or for population surveillance. Short methods are particularly useful in contexts where it is not necessary to analyse in detail the quantitative aspects of a diet. Hence, these methods investigating only a specific component of a diet may play an important role in dietary monitoring in clinical settings, health promotion, and education.

## Conclusion

This short overview has raised several questions: How to manage diet in cancer patients? How to train and support a correct food choice in cancer survivors? We argue that the answer to these questions lies in a tailored nutrition intervention. The food intake depends on different mechanisms that must be considered in any health programme: motivation, attitude, social influence, perceived behaviour control, and finally personal norms and beliefs. In order to make a healthy food choice, a personalized dietary assessment method is required. The assessment of nutrition habits and the implementation of strategies aimed at promoting healthier food-choices must be considered within a general clinical approach intended to improve patients’ QoL (see Table 2).

### Personalized nutrition method

The relevance of the nutritional issue has led to a collective mobilization to create and to promote educational programmes and guidelines, which can monitor cancer risk related to food intake and weight problems.

The psychological approach indicates that it is necessary to specify the target population, understanding related needs, values, and psychosocial demands, in order to develop a personalized programme. This is the only way to support concrete actions to prevent and to monitor chronic diseases, including obesity and weight problems in childhood and adult population.

Using a personalized approach implies adopting methods and strategies able to tailor interventions to the individual [46, 47]. This aim requires that specific demands, needs, and personal values, as well as contextual factors and clinical practice, are taken into consideration. To implement a real personalized approach, we need to first find ‘person tools’, that is, instruments that a patient may use on a day-to-day basis with a full understanding of their functions and goals based on one’s own perspective and motivation. In this sense, the personalization of medicine and, in particular, of behavioural interventions requires that the individual takes the responsibility. These instruments should be interactive, portable, and easy to use. In particular, nutrition tools should be developed as part of a more general ‘personal health records’ which aim to track an individuals’ health history and to improve their behaviour, both to prevent diseases and to enhance the QoL.

In this way, it is possible to tailor an intervention on the specific requirement of a patient. Personalization must be integrated within the general approach to a clinical setting. This means that the use of personalized instruments, for example, to assess dietary style, must be approached in a natural way by the physician in his/her day-to-day activity. Thus, these instruments and methods should be physician-friendly. The patient must also be involved in this strategy. Personalization implies patient participation, since they are invited to be an active part of their treatment, and not just a passive observer. The risk is that personalized approach will fail due to lack of responsibility sharing and/or patient commitment. Indeed, many dietary monitoring and change programmes fail due to a lack of commitment.

Increasing individual nutritional awareness is necessary to deliver comprehensive care to healthy individuals as well as to cancer patients. In general, it important to develop the specific education programmes aimed to increase this awareness, allowing an improvement in the personal commitment. In particular, programmes targeted at cancer patients are needed, since nutritional awareness seems to be particularly scarce in this population [48].

### Need analysis

A fundamental step to implement a personalized approach is the need analysis. Indeed, it is not enough to collect data about dietary habits of a patient, but we also need to know their psychological and the social background.

In order to analyse patients’ needs, an oncologist should use instruments focused on the following:

- Psychological demands
- Health beliefs and myths
- Psycho-social context
- Cognitive profiles

At present, there are no standardized instruments to achieve this vital and complex goal. Physicians must rely on more general instruments or ad-hoc methods. The cognitive science needs to be integrated with the clinical evidence to build easy-to-use and scientifically tested instruments in this area.

### Implementation of real-time and portable instruments

As already stated, the use of personalized strategies requires easy-to-use instruments for both the oncologist and the patient. The frontier of this approach is given by the technological development of electronic, portable devices with high usability, real-time features, and interactive interfaces.

Only in this way will patients actually become empowered, because he/she will have the possibility to monitor food intake, interact in real time with hospital structures, and use automatic or on-demand feedback to adjust their behaviour.

In this context, physicians would be able to monitor the ongoing situation, with respect to dietary behaviour, using simple applications for computers or other devices already available and used in clinical settings.

In particular, it would be fundamental to integrate a dietary monitoring tool within a general electronic personal health record. In this way, both physicians and patients could have at a glance the whole situation, allowing possible real-time interventions with little effort.

In conclusion, we suggest that in order to help subjects to opt for a healthier diet to improve QoL, it is fundamental to create a personalized record to assess habit, attitude, and behaviour. The more we know about the personal world of each subject, the better we will be able to identify strategies to improve healthy food choices.

## Figures and Tables

**Table 1:** Dietary assessment methods

Name	Measure	Advantage	Disadvantage
Food frequency questionnaires (FFQ)	Frequency and size of food intake	- Easy and cheap to manage - Particularly useful in epidemiological study	- Average frequency and size of food intake during a long period of time - Recalling errors and other measurement errors - Difficult to use in clinical setting (cognitive effort required)
Diet history	Frequency of food intake and food preparation	- Meal patterns and food intake are investigated extensively	- Representativeness bias (subjects often recall and report habits more than actual behaviour).
Healthy eating index (HEI)	Measure the nutrient adequacy of the diet	- Highly contextualized - Standardized scores	- Overall diet information - Qualitative data
Food diaries	Quantities of foods consumed per day	- Providing quantitative information about food consumed during a sensible period	- Motivation biases - Reactivity effect
24-hour diaries recall	Food intake during previous 24 hours	- High literacy not required - Low reactivity effect	- Subjects may not report their food intake accurately
Short dietary assessment method	Evaluating specific range of food intake	- Contribute in helping individuals to change their diet	- Fails to detect information on the all food habits

**Table 2:** Summary of the key points

**Past**
- The pioneering research of Tannenbaum (1940) stressed the correlation between cancer and nutrition. Clinical evidence suggested the relationship between overweight, food intake, physical activity, and chronic disease. - In 1981, Doll and Peto published an important report on risk factors in cancer syndrome. This year is recognized as a starting point for research in the field of food-related diseases.
**Present**
- The cancer burden and the increase of the overweight have favoured the establishment of nutritional issue and the diffusion of healthy eating programmes aimed to prevent cancer. - To promote a better healthy eating programme, it is important to know the dietary assessment methods actually available and usable. - Scientific literature is focused on some tools and methods: FFQ, diet history, HEI, diaries, 24-hour dietary recalls, and fast dietary assessment method.
**Future**
- The main goal is to enhance a personalized nutrition method both in cancer and in healthy populations. - The use of a personalized approach requires adoption of methods and strategies tailored on individuals, taking into consideration specific demands, needs, and values, in addition to contextual factors and clinical activities. - *Needs analysis* is a milestone to achieve a tailored nutrition intervention. - Electronic and interactive (first-person) personal nutrition records must be developed in order to implement a true personalized approach.
